# Tumor Markers and Their Prognostic Value in Sinonasal ITAC/Non-ITAC

**DOI:** 10.3390/cancers15123201

**Published:** 2023-06-15

**Authors:** Julius Veuger, Nona C. Kuipers, Stefan M. Willems, Gyorgy B. Halmos

**Affiliations:** 1 Department of Otorhinolaryngology/Head and Neck Surgery, University Medical Center Groningen, University of Groningen, 9700 RB Groningen, The Netherlands; julius.veuger@gmail.com (J.V.); nonacecilekuipers@gmail.com (N.C.K.); 2 Department of Surgery, St. Antonius Hospital, 3543 AZ Utrecht, The Netherlands; 3 Department of Pathology, University Medical Center Groningen, University of Groningen, 9723 GZ Groningen, The Netherlands; s.m.willems@umcg.nl

**Keywords:** (non-)ITAC, sinonasal cancer, biomarker, prognosis, survival

## Abstract

**Simple Summary:**

Prognostic markers play an important role in the risk assessment and treatment of cancers in general. The aim of this systematic review was to assess the potential prognostic markers for the rare sinonasal intestinal-type adenocarcinoma. The results from this paper may help specialists to better understand the risks of this disease and provide more insight into the prognosis. We found twenty-one biomarkers. Whilst some had a significant negative effect on prognosis, none had a positive effect. Furthermore, the biomarkers found were analyzed within the hallmarks of cancer to provide more information considering the carcinogenesis of this carcinoma. This could help in the better treatment of sinonasal intestinal-type adenocarcinoma and better patient outcomes.

**Abstract:**

One of the rare tumor entities present in the nose and paranasal sinuses is sinonasal (non-) intestinal-type adenocarcinoma (ITAC/non-ITAC). Currently, surgery with postoperative radiotherapy is the cornerstone of the treatment of these tumors. Systemic treatment is usually applied in a palliative setting. The prognosis of these tumors is very diverse. Biomarkers that may have prognostic value in these rare malignancies could help clinicians in decision-making. A systematic search of the literature was performed using the PubMed database. All studies investigating the prognostic significance of biomarkers in paranasal sinus ITAC/non-ITAC were retrieved. The findings were categorized within the hallmarks of cancer, to gain an understanding of the functions of possible prognostic biomarkers in the development of ITAC/non-ITAC. There were twenty-one studies reporting on twenty-one possible biomarkers included in the review. The expression of Mucin antigen sialosyl-Tn, C-erbB-2 oncoprotein, TIMP3 methylation, TP53, VEGF, ANXA2, MUC1 and the mucinous histological subtype were found to have a significant negative effect on survival. None of the biomarkers were found to have a positive effect on prognosis. The hallmarks ‘activating invasion and metastasis’ and ‘sustaining proliferative signaling’ seem to play the largest role in sinonasal (non-)ITAC. It could be concluded that there are multiple biomarkers foreboding a negative prognosis for ITAC/non-ITAC patients.

## 1. Introduction

Sinonasal (non-)intestinal-type adenocarcinoma (ITAC/non-ITAC) is a rare subtype of adenocarcinoma. On average, 4.6% of sinonasal adenocarcinomas are intestinal type, this clearly shows the rarity of this tumor. The diagnosis ‘ITAC’ is strongly associated with wood dust exposure and, in the majority of the cases (85%), sinonasal ITACs are located in the ethmoid sinus and the upper part of the nasal cavity. When this carcinoma presents itself in other parts of the nasal cavity, it is in most cases not related to wood dust exposure [1]. According to the SEER database, the incidence of sinonasal adenocarcinoma is 0.44 per million [2]. The five-year survival rate of sinonasal ITACs ranges from around 40 to 70%, with local recurrence as the most common type of death [3,4]. Contrary to well-researched and more common types of cancer, there is still little known about biomarkers predicting the prognosis in sinonasal ITAC/non-ITACs. Today, there is no specific treatment strategy applied for ITAC/non-ITAC sinonasal tumors. Current treatment regiments are adopted from other histological entities and uniform policy is applied for all types of malignancies of the nose and paranasal sinuses. However, histologically different tumors are biologically very different and show distinct behavior. For the current treatment of sinonasal (non-)ITACs, surgery is the cornerstone. However, as in many other cancer types, treatment is most often multimodal, with radio- and chemotherapy being a substantial part of the treatment as well. In most cases, surgery is first performed with the goal of complete surgical resection of the tumor. However, achieving clear surgical margins is in most cases not possible due to the anatomical situation. That is why surgery is often followed by local adjuvant radiotherapy [5], without any information on the radio sensitivity of these tumors. Recently, new systemic treatment strategies (e.g., targeted therapy, immunotherapy) have been introduced for other cancer types, but not in rare cancers, such as sinonasal ITAC/non-ITACs. However, in terms of other therapeutic possibilities, targeting certain proteins or genes has not been well researched in preclinical studies. Finding evidence for new treatment strategies is hampered by the rarity of these cancers, as sufficient patient inclusion for randomized controlled trials, even in a multicenter setup, is difficult. Some rare sinonasal malignancies are treated through more tumor-specific approaches; however, these treatment strategies are taken over from other tumor sites. For instance, today, sinonasal mucosal melanoma is also treated by immunotherapy, which was originally developed for cutaneous melanoma.

Therefore, the primary aim of this systematic review was to review biomarkers that may have prognostic value in intestinal-type and non-intestinal-type adenocarcinoma.

## 2. Materials and Methods

Studies were sought in the electronic database PubMed. The final search date to identify relevant studies was 18 October 2022. A search strategy was developed in cooperation with an information specialist. The search strategy utilized a combination of vocabulary and keywords focused around the terms ‘paranasal sinuses’, ‘biomarker’, ‘prognosis’ and multiple rare carcinomas. For the full search, see Appendix A. Papers were eligible for inclusion when the following criteria were met: sample size of at least 5 patients, tumor site involving the nose and paranasal sinuses, tumors of one of the following carcinomas: squamous cell carcinoma, lymphoepithelial carcinoma, sinonasal undifferentiated carcinoma (SNUC), salivary gland type carcinoma, neuroendocrine carcinoma, oncocytic carcinoma. Furthermore, only papers from the last 31 years were eligible for inclusion. Papers not containing original research, only abstracts, conference proceedings and reviews, case studies and papers not written in English were also excluded. Applying the described inclusion and exclusion criteria, the results were screened by two independent researchers (NK and JV) according to the PRISMA guidelines [6]. First, the titles and abstracts were checked. If an abstract did not provide sufficient information or the researchers did not agree on whether to include or exclude the paper, the full text was checked. After this, the full-text articles were screened. Lastly, the references of the included studies were also checked for possible inclusion. In case of discrepancy of inclusion, a consensus was always achieved with the inclusion of one of the senior authors (GH and SW). After analyzing the studies that met the inclusion criteria, it was decided to focus on papers involving ITAC/non-ITACs.

Extracted data included the number of patients, biomarkers and corresponding outcomes. Variables were: epigenetics, DNA, mRNA, proteins and others such as microvessel density and tumor budding. Relevant outcomes were overall survival, disease-free survival, disease-specific survival, hazard ratios and recurrence rates. After the extraction of relevant data, markers were analyzed according to the hallmarks of cancer [7,8]. In the case that a biomarker could be assigned to multiple hallmarks, the hallmark with the strongest indication was chosen based on the current literature. Biorender was used to present the extracted data.

To assess the quality of the included studies, the “Newcastle-Ottawa Scale (NOS) for assessing the Quality of Nonrandomized Studies” was used; see Appendix B [9].

## 3. Results

**Figure 1:**
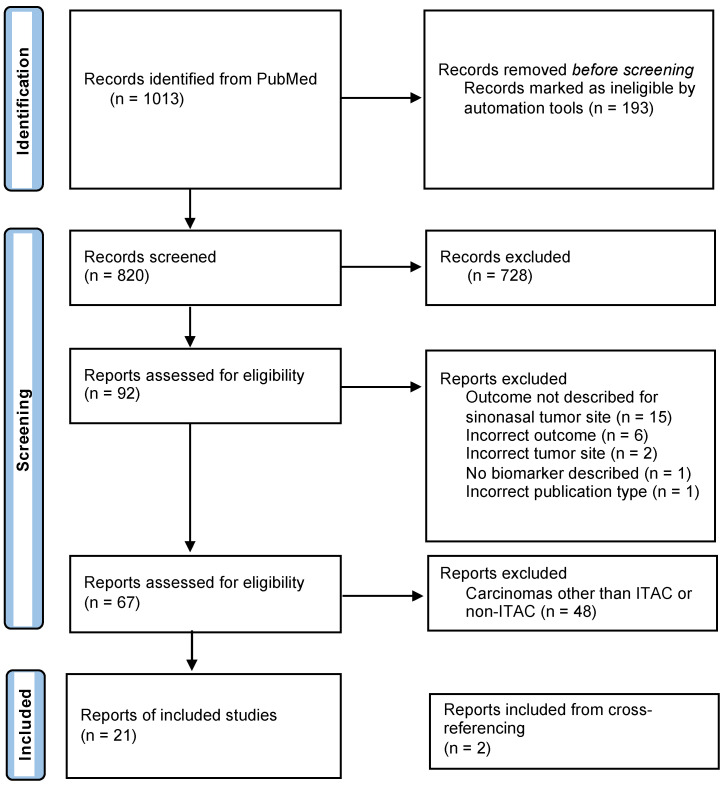
Flow diagram of study selection.

The systematic search in PubMed resulted in a total of 1013 articles (Figure 1). After the removal of duplicates and title and abstract screening, 92 articles remained. Those were reviewed in full text for eligibility. This resulted in 67 articles that met our inclusion criteria. The main reasons for exclusion were the site of the tumor and not describing prognostic data for the biomarker. The 48 studies that did not focus on ITAC/non-ITACs were also excluded, resulting in the inclusion of 19 studies. Finally, two studies were added after the cross-referencing check, which brought the total of included studies up to twenty-one. All included studies were retrospective.

The included studies are summarized in Table 1. The study size varied from 18 to 126 patients. Twenty-one different biomarkers were described within the studies, from high copy number alteration to the overexpression of certain proteins. Only EGFR, TP53 and DNA copy number alterations were described in more than one study. Of all the biomarkers found, none resulted in a significant positive effect on survival. The expression of Mucin antigen sialosyl-Tn, C-erbB-2 oncoprotein, ANXA2, MUC1, TIMP2 and TIMP3 had a negative effect on survival [10,11,12,13,14,15]. Table 2 summarizes the identified tumor markers according to the level of action.

The expression of VEGF, beta-catenin, IDH2, MUC2, MET and EGFR had no significant effect on patient outcomes [13,17,18,19,26,28]. However, in another study where the EGFR pathway was analyzed in ITACs, a mutation in EGFR mRNA was correlated with lower overall survival [16]. Mutations in TP53 were found to lead to a significant negative effect on disease-free as well as overall survival [21,22]. No association with survival was found for mutations in KRAS and BRAF [16].

With more than three copy number alterations, the effect on prognosis was poor compared to less than three copy number alterations [23]. This effect was also seen in gains at 6p22, 3q28-29, 1q22, 1q22-23, 3q28-29 and 13q31-33; and losses at 4p15-16, 4q32-35 and 10q24 [24]. The expression of microRNA-34c and microRNA-205 are predictors of poor prognosis [25]. The effect of the tumormarkers on the investigated outcome is summarized in Table 3.

Biomarkers can be categorized into four different categories: epigenetics, DNA, mRNA and proteins. None of the studies focused on epigenetic biomarkers. Most of the biomarkers were changes in the DNA. Tumor budding, a mucinous histological subtype and higher microvessel density were shown to be adverse prognostic markers in ITAC. These biomarkers did not fit into any of the previously stated categories.

**Figure 2:**
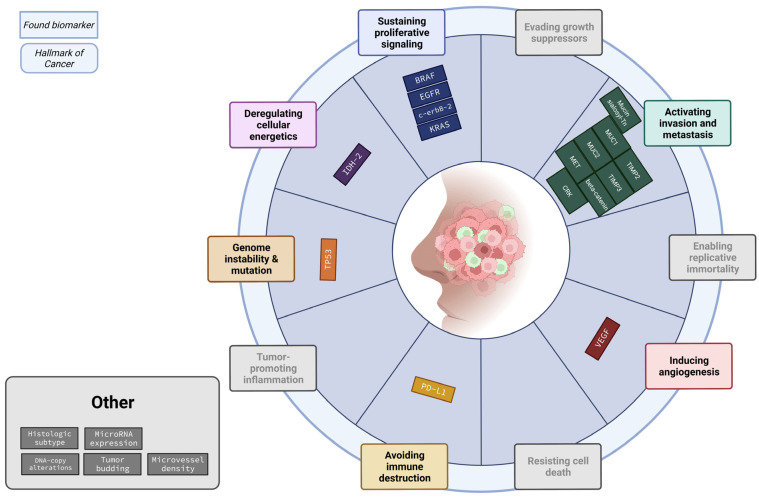
Summary of biomarkers categorized according to the hallmarks of cancer. Most of the found biomarkers fall within “Activating invasion and metastasis” and “Sustaining proliferative signaling”.

Most of the biomarkers are known biomarkers within the hallmarks of cancer. The “hallmarks of cancer” is a principle that organizes the development of cancers based on the cellular changes acquired during cancerogenesis [8]. This gives a clearer view of the complex progress of cancer development. Therefore, we classified the role of the biomarkers per hallmark. Absent from Table 4 are the hallmarks ‘Enabling replicative immortality’, ‘resisting cell death’, ‘Evading growth suppressors’ and ‘tumor-promoting inflammation’, as no biomarker was found within these hallmarks. The pathway most commonly found to play a role in the development of ITACs was the Ras-Raf-MEK-ERK pathway. KRAS, BRAF, EGFR, erbB-2 and MET all play a role in the same pathway. Notably, even though these biomarkers are mostly part of the same pathway, only the erbB-2 study found significant results. In Figure 2, the possible role of every biomarker per hallmark is visualized.

## 4. Discussion

This is the first systematic review analyzing prognostic biomarkers in sinonasal ITAC/non-ITAC. Twenty-one possible biomarkers were identified in twenty-one papers regarding prognosis in ITAC/non-ITAC. Mucin antigen sialosyl-Tn, C-erbB-2 oncoprotein, TIMP3 methylation, TP53, VEGF, ANXA2, MUC1 and the mucinous histological subtype were found to be a negative prognosticator of survival. None of the biomarkers were found to have a significant positive effect on the prognosis. Most studies had researched two of the hallmarks of cancer; namely, ‘Sustaining proliferative signaling’ and ‘Activating invasion and metastasis’.

A clinical staging system is developed to predict the prognosis of a disease; however, it is mostly based on anatomical landmarks and not on characteristics that are related to the biology of the disease [31]. An exception is the involvement of the viral status of oropharyngeal cancer in the eighth edition of the AJCC classification [31]. However, other molecular tumor features that may play an important role in prognosis are not considered in different treatment protocols. We believe researching these possible prognostic biomarkers can aid in advancing treatment protocols.

Within head and neck cancer, a significant number of prognostic biomarkers are already known. Prognostic biomarkers that have been described to influence the prognosis for other carcinomas of the head and neck often include tumor suppressor genes such as the p53 gene [32,33], oncogenes such as the EGFR gene and upregulated proteins commonly found in carcinomas, such as carbonic anhydrase expression [34], miR-21 expression [35] and programmed death ligand-1 expression [36,37]. The latter is also a well-researched target for therapy, as PD-L1 is commonly upregulated in certain types of cancers due to its ability to alter the immune response to the tumor [38]. As little is known about these biomarkers, specifically for ITAC/non-ITAC, a systematic review could direct clinicians toward a better understanding of the factors influencing prognosis. It could have clinical consequences for choosing treatment regimens and also in informing patients on the chances of survival, which may be essential in shared decision-making.

None of the found biomarkers had a positive effect on outcome. For head and neck carcinomas in general, there have been multiple studies that have found biomarkers that did have a positive effect on the outcome. An example of this is p16, which was found to have a positive effect on survival in squamous cell carcinoma of the head and neck region and positively influence the effects of radiotherapy during treatment [39,40]. Bcl-2-positivity also results in a better response to radiotherapy in head and neck squamous cell carcinoma [41]. None of the papers discussed either p16 or Bcl-2. For KRAS, however, there was a paper found discussing its effect on the outcome. This study found no correlation between KRAS and outcome. For KRAS, one other paper found that a mutation in KRAS positively impacts the response of squamous head and neck carcinoma to cetuximab [42]. These biomarkers could also potentially give promising results for sinonasal (non-)ITACs and help in the treatment of these carcinomas.

In many cancers, EGFR plays a major role in the development of these carcinomas and is often regarded as an important biomarker for a negative prognosis. In invasive squamous cell carcinoma of the head and neck region, 80% of carcinomas have an overexpression of EGFR [43]. In combination with unfavorable clinical outcomes at higher levels of expression, this has caused EGFR to be a much-studied biomarker for targeted therapies. The most prominent example of this is cetuximab, an antibody against EGFR, which is already currently being used as an effective drug to prolong overall and disease-free survival [44]. Tyrosine kinase inhibitors (TKIs) also seem to yield promising results. An example of this is erlotinib, for which Bauman et al. found that brief exposure to erlotinib alone or erlotinib combined with dasatinib significantly decreased tumor size for operable HNSCC when compared to dasatinib alone or a placebo [45].

We found three studies that had researched the effects of EGFR on prognosis. In one of them, no correlation was found [18]; in another, there was some effect on the outcome, but the findings were not significant [17]. In the third study, there was a significant correlation between EGFR mRNA and overall survival, but not between outcome and EGFR in general [16]. This could mean that the role of EGFR in sinonasal (non-)ITAC is not as significant as in some other head and neck carcinomas. Since the role of EGFR in sinonasal (non-)ITACs is not completely clear yet, further research should clarify whether EGFR-targeted therapy is a viable option for sinonasal (non-)ITAC.

As mentioned before, PD-L1 is a well-established biomarker. Its overlapping hallmark is avoiding immune destruction. Riobello et al. researched this protein as a possible marker for targeted therapy in ITACs [27]. They found no significant difference in prognostic data for multivariate analysis correcting for disease stage and histological subtype. This study concludes that due to limited treatment options available, it could serve as a therapeutic target, as 33% of the tumor cells expressed PD-L1. This is not uncommon. A recent study by Blatt et al. researched the role of PD-L1 expression between oral and oropharyngeal squamous cell carcinoma. It concluded that no association between PD-L1 expression and patient outcome could be found [46].

We found a profound difference in the amount and variance between known biomarkers for other head and neck carcinomas and that for sinonasal (non-)ITACs. For instance, a meta-analysis discussing oral tongue squamous cell carcinoma, found 184 biomarkers. The most assessed biomarkers for oral tongue squamous cell carcinoma were p53, Ki-67, p16, VEGFs and cyclin D1, with the best results for cyclin D1 [33]. Of these biomarkers, only p53 and VEGF were assessed for sinonasal (non-ITACs). Research into the prognostic role of cyclin D1 in sinonasal (non-)ITACs could possibly give promising results for this carcinoma. For resectable esophageal adenocarcinoma, 82 prognostic biomarkers have been identified [37]. Some of these, such as EGFR, beta-catenin, MUC2 and c-erbB2, were also identified for sinonasal (non-)ITACs. The number of biomarkers is in sharp contrast with the meager twenty-one possible biomarkers we found. The most promising biomarkers for both esophageal adenocarcinoma and oral tongue squamous cell carcinoma have not yet been researched in sinonasal ITACs.

The hallmark of cancer most often associated with sinonasal (non-)ITACs is the hallmark ‘activating invasion and metastasis’. A total of eight biomarkers are associated with this hallmark. The fact that this hallmark plays a significant role in the development of this carcinoma could possibly explain its aggressive nature. The hallmark second most often associated with sinonasal (non-)ITACs is the hallmark ‘sustaining proliferative signaling’, with a total of four biomarkers.

The conclusions from the found studies were not always in coherence with what would be expected based on the general role of the biomarker in cancerogenesis. This could mean these biomarkers do not play a major role in cancerogenesis for sinonasal ITACs. Examples of this are the study results from the studies regarding EGFR [16,17,18] and PD-L1 [27]. In head and neck carcinomas and cancers in general, the mechanisms, significance as a biomarker and possible targeted therapies for these biomarkers have been widely studied. The results of this systematic review show that these biomarkers most likely do not play a significant role in the prognosis of (non-)sinonasal ITACs.

As ITACs/non-ITACs are rare entities, the found studies often drew conclusions regarding a group of sinonasal carcinomas including ITACs/non-ITACs. They were often researched together with SCC [15], adenoid cystic carcinomas [13,28] and SNUC [19]. A meta-analysis could not be performed due to the heterogeneity of the reported outcomes, devaluating this systematic review. It would be wise to update the review when more biomarkers have been researched. Notably, almost all studies were determined to be of good quality according to the NOS. Further research on the molecular properties of ITACs/non-ITACs is hampered by the rarity of these tumors. A logical next step would be analyzing the biomarkers from open public databases.

## 5. Conclusions

In total, we found twenty-one papers that studied the effect of biomarkers on the prognosis of sinonasal (non-)ITACs. This resulted in a total of twenty-one biomarkers.

The expression of Mucin antigen sialosyl-Tn, C-erbB-2 oncoprotein, TIMP3 methylation, TP53, VEGF, ANXA2, MUC1 and the mucinous histological subtype had a significant negative effect on survival. For ITACs/non-ITACs, the most well-researched pathway is the Ras-Raf-MEK-ERK. No biomarkers were found to have a positive effect on prognosis. The hallmarks most often associated with sinonasal (non-)ITACs are the hallmarks of ‘activating invasion and metastasis’ and ‘sustaining proliferative signaling’. A thorough understanding of the biomarkers involved in ITAC/non-ITAC prognosis could provide therapeutic targets for enhanced treatment options.

## Figures and Tables

**Table 1:** Summary of included studies.

Study: PMID; Autor; Year; Included Patients	Tumor Type	Tumormarker	Measured Outcome	Effect on Outcome	Significant Findings	Quality Assessment
23791006; Projetti, 2013 (*N* = 39) [16]	ITAC	EGFR variant mRNA expression EGFR mutation KRAS mutation BRAF mutation	Overall survival	Negative	EGFR mRNA: *p* = 0.03 No association with EGFR, KRAS or BRAF mutation	High Quality
19213595; Franchi, 2009 (*N* = 18) [17]	ITAC	EGFR overexpression	Disease-free and overall survival	Negative	DFS: *p* = 0.57 OS: *p* = 0.62	High Quality
23055340; García-Inclán, 2012 (*N* = 98) [18]	ITAC	EGFR protein overexpression	Recurrence, metastasis and survival	-	No correlation found	High Quality
27107016; Perez-Escuredo, 2016 (*N* = 37) [14]	ITAC	Multiple recurrent genetic alterations deletion of TIMP2 deletion of CRK	Overall survival	Negative	Loss at 4q32-ter: *p* = 0.000 Gain at 6p22: *p* = 0.008 Gain at 3q29: *p* = 0.025 Gain at 1q22: *p* = 0.028 Loss at TIMP2: *p* = 0.022 Loss at CRK: *p* = 0.045	High Quality
31876581; Riobello, 2020 (*N* = 52) [19]	(n-)ITAC + SNUC + ONB + SCC + SNEC	IDH2 mutation	Disease-specific survival	-	Not enough IDH2 positive (n-)ITAC cases	High Risk
24913906; Projetti, 2015 (*N* = 72) [20]	ITAC	MET protein levels gene copy number	Progression-free survival and overall survival	-	No correlation found	High Risk
27301901; Costales, 2016 (*N* = 50) [15]	ITAC + SCC	TIMP3 methylation	Overall survival, disease-free survival, metastasis and recurrence	Negative	OS: *p* = 0.027 DFS: *p* = 0.001 Metastasis: *p* = 0.005 Recurrence: *p* = 0.005	High Quality
23369851; Bossi, 2013 (*N* = 74) [21]	ITAC	TP53 mutation	Overall and disease-free survival after neoadjuvant chemotherapy	Negative	OS: *p* = 0.023 DFS: *p* = 0.010	High Quality
15611505; Licitra, 2004 (*N* = 30) [22]	ITAC	TP53 mutation	Complete remission after primary chemotherapy	Negative	*p* < 0.0001	High Quality
19073009; Hermsen, 2009 (*N* = 22) [23]	ITAC	High DNA copy number alterations (CNA)	Metastasis, intracranial invasion, overall survival, recurrence	Negative	Intracranial invasion: *p* = 0.038 Recurrence: *p* = 0.387 Metastasis: *p* = 0.662 OS: *p* = 0.002	High Risk
28963820; López-Hernández, 2018 (*N* = 96) [24]	ITAC	Copy number alterations	Overall survival	Negative	Gain at 1q22-23: *p* = 0.001 Gain at 3q28-29: *p* = 0.016 Gain at 6p22: *p* = 0.000 Gain at 13q31-33: *p* = 0.031 Loss at 4p15-16: *p* = 0.000 Loss at 4q32-35: *p* = 0.000 Loss at 10q24: *p* = 0.011	High Quality
34647653; Re, 2022 (*N* = 43) [25]	ITAC	miR-205 expression miR-34c expression miR-449a expression miR-192 expression	Disease-free survival, Overall survival	Negative	DFS: miR-205 *p* = 0.034 miR-34c *p* = 0.034 miR-449a *p* = 0.013 miR-192 not significant OS: miR-205 *p* = 0.0005 miR-34c *p* = 0.023 miR-449a and miR-192 not significant	High Quality
20970165; Rodrigo, 2011 (*N* = 57) [12]	ITAC	ANXA2 expression	Disease-specific survival	Negative	*p* = 0.004	High Quality
22125792; Díaz-Molina, 2011 (*N* = 83) [26]	ITAC	Beta-catenin expression	Overall survival	Negative	*p* = 0.054	High Quality
9570628; Gallo, 1998 (*N* = 28) [11]	ITAC	C-erbB-2 oncoprotein expression	5-year disease-free and overall survival curves	Negative	5-year DFS: *p* = 0.02 OS: *p* = 0.07	High Quality
8736175; Franchi, 1996 (*N* = 30) [10]	ITAC	Mucin antigen sialosyl-Tn expression	5-year survival rate and disease-free interval	Negative	5-year survival: *p* = 0.0001 disease-free interval: *p* = 0.0001	High Quality
31076280; Taverna, 2019 (*N* = 66) [13]	(n-)ITAC + adenoid cystic carcinoma	MUC1 expression MUC2 expression	Overall survival	Negative	MUC1: *p* = 0.05 MUC2: *p* = 0.4	High Quality
29356178; Riobello, 2018 (*N* = 126) [27]	ITAC + SCC	PD-L1	Overall survival, disease-specific survival, disease-free survival	-	OS: *p* = 0.692 DSS: *p* = 0.918 DFS: *p* = 0.146	High Quality
16564912; Valenta, 2006 (*N* = 105 of mixed tumor types) [28]	ITAC + SCC + adenoid cystic carcinoma	Microvessel density VEGF expression	5-year disease-free survival	Negative	Microvessel density: mortality hazard ratio = 1.33 VEGF: *p* = 0.06	High Risk
21668475; Franchi, 2011 (*N* = 62) [29]	ITAC	Histological subtype	Disease-free interval and disease-free survival	Negative for mucinous type	Disease-free interval: *p* = 0.005 DFS: *p* < 0.001	High Risk
31980958; Maffeis, 2020 (*N* = 32) [30]	ITAC	Tumor budding	Overall survival, disease-free survival, lymphovascular invasion, recurrence and death of disease	Negative	OS: *p* = 0.013 DFS: *p* = 0.0002 Lymphovascular invasion: *p* = 0.008 Recurrence: *p* = 0.0005 Death of disease: *p* = 0.02	High Quality
total number of patients: 1220						

OS: overall survival; DFS: disease-free survival; DSS: disease-specific survival; SCC: squamous cell carcinoma; SNUC: sinonasal undifferentiated carcinoma; ONB: olfactory neuroblastoma; SNEC: small cell neuroendocrine carcinoma.

**Table 2:** Summary of the identified tumor markers according to the level of action.

Subdivision	Markers
Epigenetics	None
DNA	BRAF, CRK, EGFR, IDH2, KRAS, MET, TIMP2, TIMP3, TP53, DNA copy number alterations
RNA	microRNA-34c, microRNA-192, microRNA-205, microRNA-449a
Proteins	ANXA2, beta-catenin, c-erbB-2, EGFR, MET, mucin sialosyl-Tn, MUC1/MUC2, PD-L1, VEGF
Other	Histological subtype, microvessel density, tumor budding

**Table 3:** Summary of the described outcomes and their prognostic value of each identified tumor marker.

Tumor Marker	Described Outcome	Prognosis	References (PMID)
BRAF	Survival	No difference	23791006 [16]
CRK	Overall survival	Poor in case of loss	27107016 [14]
EGFR	Disease-free and overall survival, recurrence and metastasis	No difference	19213595 [17] 23791006 [16] 23055340 [18]
IDH2	Disease-specific survival and disease-free survival	-	31876581 [19]
KRAS	Survival	No difference	23791006 [16]
MET	Progression-free survival and overall survival	No difference	24913906 [20]
TIMP2	Overall survival	Poor in case of loss	27107016 [14]
TIMP3	Overall survival, disease-free survival	Poor	27301901 [15]
TP53	Survival, effect on chemotherapy	Poor if mutated	15611505 [22] 23369851 [21]
DNA copy number alterations (CNA)	Metastasis, intracranial invasion, mean overall survival, recurrence	Poor if high CNA	19073009 [23]
DNA copy number alterations (CNA)	Overall survival	4q32-ter: poor in case of loss 6p22: poor in case of gain 3q29: poor in case of gain 1q22: poor in case of gain	27107016 [14] 28963820 [24]
MicroRNA expression	Disease-free and overall survival	Poor	34647653 [25]
ANXA2 expression	Disease-specific survival	Poor	20970165 [12]
Beta-catenin	Overall survival	Poor	22125792 [26]
c-erbB-2 oncoprotein	Disease-free and overall survival	Poor	9570628 [11]
Mucin antigen sialosyl-Tn	5-year survival	Poor	8736175 [10]
MUC1/MUC2	Overall survival	Poor	31076280 [13]
PD-L1	Disease-specific, overall and disease-free (1 year) survival	Poor	29356178 [27]
VEGF	Mortality, clinical stage, histological grading	Poor	16564912 [28]
Histological subtype	Disease-free interval and disease-free survival	Poor in case of mucinous subtype (expression of MUC 1 and MUC2)	21668475 [29] 31076280 [13]
Microvessel density	Mortality, clinical stage, histological grading	Poor	16564912 [28]
Tumor budding	Disease-free and overall survival	Poor	31980958 [30]

**Table 4:** Biomarkers within hallmarks of cancer and their role in the cancer cascade.

Hallmark	Biomarker	Its Role within the Cascade That Leads to Cancer	Conclusions Drawn from Our Selected Studies PMID Numbers
Sustaining proliferative signaling	KRAS	Downstream of receptor tyrosine-kinases (RTK). Ras activates the Ras-Raf-MEK-ERK pathway and the PI3K pathway.	KRAS, BRAF or EGFR mutations not associated with progression-free survival (PFS) and overall survival (OS) 23791006 [16] EGFR overexpression not associated with disease-free and overall survival. Furthermore, no association between T stage, lymph node metastasis or distant metastasis. 19213595 [17]
	BRAF	BRAF is part of the Ras-Raf-MEK-ERK pathway and activates MAP2K1/MAP2K2.	
	EGFR (HER1)	EGFR is a transmembrane RTK which mainly signals through the Ras-Raf-MEK-ERK pathway	
	erbB-2 (HER2)	erbB-2 is a transmembrane RTK which mainly signals through the Ras-Raf-MEK-ERK pathway	Significant correlation between c-erbB-2 expression and 5-year disease-free survival (*p* = 0.02), overall survival (*p* = 0.07) and distant metastases (*p* = 0.08) by univariate analysis. However, by multivariate analysis, only disease-free survival (*p* = 0.046) is significant. 9570628 [11]
Evading growth suppressors	N/A	N/A	N/A
Activating invasion and metastasis	CRK (loss of CRK at 17p13)	Important scaffolding protein in downstream RTK signaling through Src family tyrosine kinases. Stimulates the activation loop of intracellular signaling.	CRK copy number loss is associated with significantly worse overall survival. 27107016 [14]
	MET	RTK signals through Ras and PI3K pathways and promotes proliferation, migration and invasion.	MET protein levels and MET gene copy numbers not associated with survival 24913906 [20]
	TIMP2	TIMP2 blocks the activity of matrix metalloproteinases (in particular MMP-9), which promotes malignant outgrowth. Protects the extracellular matrix of tumors from degradation by a disintegrin and metalloproteinase	TIMP2 mutation with loss of function associated with lower overall survival (*p* = 0.022) 27107016 [14]
	TIMP3	TIMP3 blocks the activity of matrix metalloproteinases, which promote malignant outgrowth. Protects the extracellular matrix of tumors from degradation by a disintegrin and metalloproteinase	Methylation of TIMP3 significantly associated with worse disease-free (*p* = 0.027) and overall survival (*p* = 0.001) 27301901 [15]
	beta-catenin	Downstream in Wnt-pathway	In 31% of patients with ITACs, nuclear β-catenin is present, thus Wnt-pathway is active and conveys a worse prognosis. 22125792 [26]
	mucin sialosyl-Tn	Mucin antigen, formed by incomplete glycosylation of a mucin glycoprotein	The 5-year survival rate and disease-free interval of patients with S-Tn-positive adenocarcinomas were significantly lower than those with negative adenocarcinomas (17.8% versus 72%, *p* = 0.0001; 16.6% versus 40%, *p* = 0.0001, respectively). 8736175 [10]
	MUC1	Membrane-bound protein that plays an essential role in forming protective mucous barriers on epithelial surfaces and in intracellular signaling	In the group of ITACs, MUC1 expression was associated with shorter overall survival (*p* = 0.05) 31076280 [13]
	MUC2	Membrane-bound protein that plays an essential role in forming protective mucous barriers on epithelial surfaces and in intracellular signaling	Overall survival was not related to MUC2 expression (*p* = 0.4) 31076280 [13]
Enabling replicative immortality	N/A	N/A	N/A
Inducing angiogenesis	VEGF	VEGF-A binds to VEGFR-1 and VEGFR-2, and regulates endothelial cell proliferation, migration, vascular permeability, secretion.	No results on ITACs alone, but in a group of tumors consisting of ITACs and SCC, no significant correlation between VEGF positivity and prognosis. 16564912 [28]
Resisting cell death	N/A	N/A	N/A
Avoiding immune destruction	PD-L1	PD-L1 binds to PD-1, which leads to inhibition of T-cell activation and cytokine production and subsequent immune escape of tumor cells.	Significant difference in 1-year disease-free survival in tumors with higher membranous PD-L1 expression. 29356178 [27]
Tumor promoting inflammation	N/A	N/A	N/A
Genome instability and mutation	TP53	Tumor suppressor gene plays a role in and leads to a cell cycle arrest upon DNA damage.	Mutation of TP53 associated with less response to chemotherapy, and thereby, worse survival. 15611505 [22] Mutation of TP53 significantly associated with worse overall survival. 23369851 [21]
Deregulating cellular energetics	IDH2	Plays an important role in the TCA cycle.	Only 1/48 ITACs with mutant IDH. 31876581 [19]

## Data Availability

No new data were created or analyzed in this study. Data sharing is not applicable to this article.

## Appendix A

(“Carcinoma, Squamous Cell”[Mesh] OR “Carcinoma, Adenosquamous”[Mesh] OR acinar cell carcinoma*[tiab] OR “Carcinoma, Small Cell”[Mesh] OR “Carcinoma, Adenoid Cystic”[Mesh] OR “Carcinoma, Acinar Cell”[Mesh] OR “Carcinoma, Mucoepidermoid”[Mesh] OR “Adenocarcinoma, Clear Cell”[Mesh] OR “Carcinoma, Neuroendocrine”[Mesh] OR verrucous carcinoma*[tiab] OR squamous cell carcinoma*[tiab] OR adenosquamous carcinoma*[tiab] OR lymphoepithelial carcinoma*[tiab] OR undifferentiated carcinoma*[tiab] OR Salivary gland type carcinoma*[tiab] OR Adenoid cystic carcinoma*[tiab] OR Acinic cell carcinoma*[tiab] OR mucoepidermoid carcinoma*[tiab] OR epithelial-myoepithelial carcinoma*[tiab] OR clear cell carcinoma*[tiab] OR myoepithelial carcinoma*[tiab] OR carcinoma ex pleomorphic adenoma*[tiab] OR polymorphous low-grade adenocarcinoma*[tiab] OR Neuroendocrine carcinoma*[tiab] OR nec[tiab] OR lcnec*[tiab] OR oncocytic carcinoma*[tiab] OR well differentiated neuroendocrine carcinoma*[tiab] OR well differentiated nec[tiab] OR Moderately differentiated carcinoma*[tiab] OR Moderately differentiated nec[tiab] OR Poorly differentiated carcinoma*[tiab] OR Poorly differentiated nec[tiab] OR “Paranasal Sinus Neoplasms”[Mesh])

AND

(“Paranasal Sinuses”[Mesh] OR Sinonasal*[tiab] OR sinus*[tiab] OR nose*[tiab] OR nasal[tiab] OR paranasal[tiab])

AND

(pathway*[tiab] OR molecule*[tiab] OR biomarker*[tiab] OR “Molecular Sequence Data”[Mesh] OR “Mutation”[Mesh] OR “Biomarkers”[Mesh] OR “Gene Expression Regulation”[Mesh] OR mutat*[tiab] OR “Immune Checkpoint Proteins”[Mesh] OR “Chemotactic Factors”[Mesh] OR “Inflammation Mediators”[Mesh] OR “Intercellular Signaling Peptides and Proteins”[Mesh] OR checkpoint*[tiab] OR gene*[tiab])

AND

(“Prognosis”[Mesh] OR prognos*[tiab] OR survival*[tiab] OR recurren*[tiab] OR progress*[tiab] OR treatment outcome*[tiab] OR predict*[tiab] OR failure*[tiab])

## Appendix B

Newcastle-Ottawa quality assessment scale cohort studies

Note: A study can be awarded a maximum of one star for each numbered item within the Selection and Outcome categories. A maximum of two stars can be given for Comparability

Selection

(1) Representativeness of the exposed cohort
  (a) truly representative of the average _______________ (describe) in the community Ø
  (b) somewhat representative of the average ______________ in the community Ø
  (c) selected group of users e.g., nurses, volunteers
  (d) no description of the derivation of the cohort
(2) Selection of the non exposed cohort
  (a) drawn from the same community as the exposed cohort Ø
  (b) drawn from a different source
  (c) no description of the derivation of the non exposed cohort
(3) Ascertainment of exposure
  (a) secure record (e.g., surgical records) Ø
  (b) structured interview Ø
  (c) written self report
  (d) no description
(4) Demonstration that outcome of interest was not present at start of study
  (a) yes Ø
  (b) no
  Comparability
(1) Comparability of cohorts on the basis of the design or analysis
  (a) study controls for _____________ (select the most important factor) Ø
  (b) study controls for any additional factor Ø (This criteria could be modified to indicate specific control for a second important factor.)
  Outcome
(1) Assessment of outcome
  (a) independent blind assessment Ø
  (b) record linkage Ø
  (c) self report
  (d) no description
(2) Was follow-up long enough for outcomes to occur
  (a) yes (select an adequate follow up period for outcome of interest) Ø
  (b) no
(3) Adequacy of follow up of cohorts
  (a) complete follow up—all subjects accounted for Ø
  (b) subjects lost to follow up unlikely to introduce bias—small number lost—> ____ % (select an adequate %) follow up, or description provided of those lost) Ø
  (c) follow up rate < ____% (select an adequate %) and no description of those lost
  (d) no statement

**Table A1:** Quality assessment of the included studies.

Study: PMID; Autor; Year (n)	Selection	Comparability	Outcome	Total
23791006; Projetti, 2013 (*N* = 39) [16]	4 (histologic subtype)/3 (KRAS, BRAF)	0	3	6/7
9570628; Gallo, 1998 (*N* = 28) [11]	4	1	3	8
19213595; Franchi, 2009 (*N* = 18) [17]	4	1	2	7
23055340; García-Inclán, 2012 (*N* = 98/65) [18]	3	1	3	7
24913906; Projetti, 2015 (*N* = 72) [20]	3	2	1	6
22125792; Díaz-Molina, 2011 (*N* = 83) [26]	4	2	3	9
27107016; Perez-Escuredo, 2016 (*N* = 37) [14]	4	0	3	7
27301901; Costales, 2016 (*N* = 50) [15]	4	1	3	8
31876581; Riobello, 2020 (*N* = 52) [19]	3	1	2	6
23369851; Bossi, 2013 (*N* = 74) [21]	4	1	3	8
15611505; Licitra, 2004 (*N* = 30) [22]	4	1	3	8
16564912; Valenta, 2006 (*N* = 105 of mixed tumor types) [28]	3	0	2	5
20970165; Rodrigo, 2011 (*N* = 57) [12]	3	1	3	7
8736175; Franchi, 1996 (*N* = 30) [10]	3	1	3	7
31076280; Taverna, 2019 (*N* = 66) [13]	4	1	3	8
19073009; Hermsen, 2009 (*N* = 22) [23]	3	1	2	6
28963820; López-Hernández, 2018 (*N* = 96) [24]	4	1	2	7
21668475; Franchi, 2011 (*N* = 62) [29]	3	1	2	6
31980958; Maffeis, 2020 (*N* = 32) [30]	4	1	3	8
29356178; Riobello, 2018 (*N* = 126) [27]	4	2	3	9
34647653; Re, 2022 (*N* = 43) [25]	4	2	3	9
